# Social Determinants of Health, the Diagnostic Odyssey, and Genetic Testing for Global Developmental Delay/Intellectual Disability: A Qualitative Study

**DOI:** 10.21203/rs.3.rs-8378360/v1

**Published:** 2026-01-28

**Authors:** Jordan J. Cole, Emily Killian, Angela D. Sellitto, Alex Ramsey, Joyce E. Balls-Berry, Christina Gurnett

**Affiliations:** University of Colorado Anschutz School of Medicine; University of Iowa College of Public Health; Washington University in St. Louis School of Medicine; Washington University in St. Louis School of Medicine; Washington University in St. Louis School of Medicine; Children’s Hospital of Philadelphia

**Keywords:** global developmental delay/intellectual disability, implementation science, genetic testing, diagnostic odyssey, health equity, ethnoracial disparities, social determinants of health

## Abstract

**Background:**

Genetic testing is recommended for all children with global developmental delay or intellectual disability (GDD/ID) without a clearly-identifiable acquired etiology, as it leads to a rare disease diagnosis up to 40% of the time and has implications for medical management and family planning. However, families often experience years-long journeys from the time of initial presentation with developmental concerns to the time of genetic testing, if it is completed at all. An implementation science-based approach to understanding determinants of genetic testing for GDD/ID is ideal for identifying key targets for future strategies to improve uptake.

**Methods:**

The Health Equity Implementation Framework was used to adapt a semi-structured qualitative interview guide from a previous study. Interviews were conducted with 18 caregivers of children with GDD/ID who had been evaluated in pediatric neurology clinics at a single tertiary care institution. Over half of participants (55.6%) self-identified with at least one historically underrepresented demographic (33.3% Black/African American, 27.8% with high school or less education, 27.8% with high neighborhood-level area deprivation, 11.1% rural). Content analysis was performed using inductive and deductive coding. Content saturation was reached.

**Results:**

Six themes reflected multilevel factors influencing uptake of genetic testing: (1) caregiver search for answers, advocacy, and empowerment; (2) real-world healthcare accessibility; (3) financial strain and insurance coverage; (4) trust and communication with providers; (5) racial disparities and discrimination; and (6) social support and community networks. Most caregivers viewed genetic testing positively. Pathways to testing were often complex and uneven. Social determinants (insurance, employment flexibility, rurality, family structure, and racism) shaped families’ ability to access timely testing and follow-up. System-level barriers such as uncoordinated referrals, inconsistent provider knowledge, and long wait times compounded these challenges. Together, these factors created unequal diagnostic journeys for families, even within the same health system.

**Conclusions:**

We identified caregiver-informed targets to improve the quality and equity of care for patients with GDD/ID including insurance coverage, diagnostic efficiency, resource availability and awareness, healthcare navigation, and cultural humility. These targets inform the development and adaptation of implementation strategies to improve uptake and reduce the protracted time of the diagnosis and genetic testing journey.

**Trial registration:**

Not applicable

## BACKGROUND

For families of children with neurodevelopmental disorders such as global developmental delay or intellectual disability (GDD/ID), the process of navigating the medical system in search of answers is typically complex and prolonged, often appropriately termed the “diagnostic odyssey.” Over the past fifteen years, an explosion in genomic knowledge and clinical availability of genetic testing has made it possible to shorten the diagnostic odyssey for many individuals with GDD/ID, (1) but uptake of genetic testing has been suboptimal. This is despite published clinical guidelines from both the American College of Medical Genetics (ACMG) and the American Academy of Pediatrics (AAP) recommending early consideration of broad genetic testing for the majority of children with GDD/ID. (2, 3) In the United States, studies have found that only about 25–40% of individuals with GDD/ID have ever received any type of genetic testing, and only about 10% have received testing in line with current academic society guidelines.(4–7) Further compounding access concerns are disparities in usage of genetic services based on social determinants of health including race/ethnicity and socioeconomic status.(4, 8–11)

Prior studies have been conducted to investigate caregiver (parent/guardian) experiences with and perspectives on genetic testing that may contribute to low testing rates, but they have not incorporated formal implementation science methodology in their planning or analyses.(12–16) Implementation science is the study of how and why novel evidence-based innovations (such as genetic testing for children with GDD/ID) are or are not successfully integrated into clinical practice.(17) Through the application of theory-based frameworks, implementation science allows practitioners and researchers to approach real-world problems and questions wholistically and pragmatically. We sought to apply an implementation science framework to understand the experiences and perspectives of caregivers of children with GDD/ID related to genetic testing and healthcare as a whole. In addition, we made efforts to recruit participants from traditionally underrepresented backgrounds in research, as prior studies investigating caregiver perspectives have primarily included non-Hispanic White, highly educated, and high income individuals. In particular, we focused our efforts on over-representation from the Black/African American community due to the demographics of the patient population in St. Louis, Missouri, where this study was conducted (15% non-Hispanic Black/African American, 78% non-Hispanic White),(18) and known systemic health disparities in the Black/African American population in the United States that have led to calls for research to reduce such inequities.(19)

## METHODS

### Study Overview

The study was approved by the Washington University in St. Louis School of Medicine’s Institutional Review Board. Informed by an implementation science lens, semi-structured qualitative interviews were used to investigate diagnostic odyssey and genetic testing perspectives/experiences of caregivers of children with GDD/ID. Clinical trial number: not applicable.

### Design and Theoretical Framework

**Figure 1:**
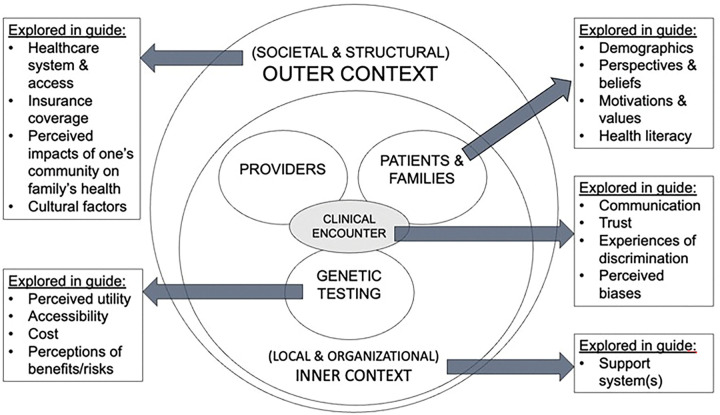
Application of the Health Equity Implementation Framework to a Semi-Structured Interview Guide. Figure 1 demonstrates a simplified version of the Health Equity Implementation Framework (HEIF)(21) as it was applied in this study. The HEIF emphasizes consideration of multilevel domains influencing uptake of an innovation. It is essential to consider aspects of the innovation itself (genetic testing) as well as characteristics of the recipients of the innovation (patients/caregivers and providers) and the interaction between all three (the clinical encounter), as well as the broader contextual environment in which all are functioning.

The methodological orientation used was directed content analysis.(20) The Health Equity Implementation Framework (HEIF)(21) was applied as a conceptual framework to inform the adaptation of the semi-structured interview guide and data analysis (Fig. 1).

Figure 1 demonstrates a simplified version of the Health Equity Implementation Framework (HEIF)(21) as it was applied in this study. The HEIF emphasizes consideration of multilevel domains influencing uptake of an innovation. It is essential to consider aspects of the innovation itself (genetic testing) as well as characteristics of the recipients of the innovation (patients/caregivers and providers) and the interaction between all three (the clinical encounter), as well as the broader contextual environment in which all are functioning.

The semi-structured interview guide (**Additional File 1**) was adapted from the Event History Calendar Interview: Diagnostic Odyssey, a tool that was developed, piloted, and used in a prior study investigating the diagnostic odyssey among African American children with autism spectrum disorder.(22) Permission from the original authors was obtained prior to adaptation. A review of existing literature on caregiver experiences/perspectives on genetic testing was also used to inform the interview guide adaptations.(23–25) The adapted version was pilot-tested in one caregiver of children with GDD/ID. The procedures reported here are in alignment with the Consolidated Criteria for Reporting Qualitative Studies (COREQ) 32-item checklist(26) (**Additional File 2**).

### Setting

The setting for this study was the catchment area of a tertiary care children’s hospital’s pediatric neurology clinics located in the St. Louis, MO metropolitan area in the United States. The interviews took place virtually by phone or video chat, with interviewers located in a private, quiet room at home or the workplace, and participants located at their site of choice.

### Eligibility

Individuals were eligible for interview participation if they were the caregiver (parent/guardian) of a child ( < = 18 years old) with GDD/ID who had been evaluated in the tertiary care hospital’s pediatric neurology clinic within the past one year. GDD/ID diagnoses were based on electronic health record (EHR) review. Individuals were excluded if they did not speak English (approximately 1% of the clinic population), if the child was in foster care, had a documented acquired etiology of GDD/ID, or had trisomy 21 diagnosed prenatally or immediately after birth.

### Recruitment

Two methods of recruitment took place between January to May 2023. First, in a convenience sampling method, flyers advertising the study were posted and distributed in the pediatric neurology clinic. The flyers contained a QR code for interested participants to provide initial information and request a recruitment call/email. They were only available in English per the study eligibility criteria. A study team member (JJC) screened interested participants’ children’s EHR to determine eligibility, and then EAK contacted the caregiver to provide more information and schedule an interview date.

Second, a purposeful sampling method was employed with the intent to increase the ethnoracial diversity of participants. All patients who had been seen in the pediatric neurology clinic between 1/15/23 to 3/15/23 with EHR-based race/ethnicity identified as Black/African American (including multiracial Black/African American) were screened for eligibility through EHR review, and those eligible were contacted by phone.

**Figure 2:**
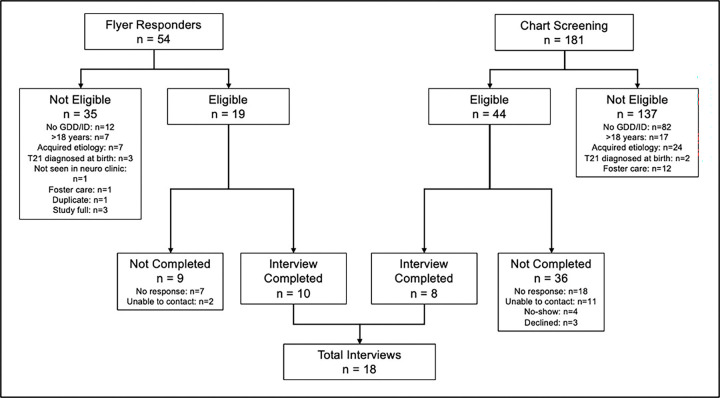
Recruitment Flowchart for Qualitative Interviews Figure 2 demonstrates the two methods of sampling used to identify eligible participants for the study, and the numbers of individuals moving through each step of recruitment/study completion. Among those with interviews not completed, “no response” indicates that a recruitment message was transmitted but no response was received, whereas “unable to contact” indicates that the individual’s contact method(s) did not accept messages.

A total of 54 responses to the flyers were received. Of responders, 19 (35.1%) were eligible, and of those eligible, 9 (47.4%) completed interviews. A total of 181 patients with EHR-based race/ethnicity identified as Black/African American were screened for eligibility. Of those screened, 44 (24.3%) were eligible, and of those eligible, 8 (18.2%) completed interviews. Reasons for ineligibility and lack of study participation are outlined in Fig. 2.

Figure 2 demonstrates the two methods of sampling used to identify eligible participants for the study, and the numbers of individuals moving through each step of recruitment/study completion. Among those with interviews not completed, “no response” indicates that a recruitment message was transmitted but no response was received, whereas “unable to contact” indicates that the individual’s contact method(s) did not accept messages.

### Participants & Patients

Interview participant (caregiver) characteristics, as well as characteristics of the patients about whom the interviews were focused, are outlined in Table 1. Patients’ specific genetic diagnoses are not included in this manuscript to protect patient privacy given the small sample size and rarity of many neurogenetic conditions. Informed consent was obtained immediately prior to the start of each interview. Race/ethnicity reported here is self-identified by the participants.

### Data Collection

Semi-structured qualitative interviews were conducted by phone or video call (as preferred by the participant) between February to June 2023. The interviews lasted approximately 45–60 minutes each and were conducted an average of 1.8 months (range 0–4 months) from the date of the last neurology clinic visit. The interviews were audio/video recorded and subsequently transcribed and deidentified for coding. In addition to the interview guide, probing questions were used to clarify participant responses.

### Data Analysis

Authors JJC and EAK developed the initial codebook using deductive codes based on the existing literature and the interview guide (informed by the Health Equity Implementation Framework), along with inductive codes generated from field notes and review of the transcripts. Both authors independently coded the first 2 interviews, then collaborated to review assignments, refine code definitions, and reach consensus on the finalized codebook (**Additional File 3)**. This process ensured reliability and validity in code application.(27, 28) Author EAK then independently coded all 18 interviews using the final codebook, allowing for consistent application of codes across the dataset. A web-based qualitative analysis software, Dedoose, was used to organize coding, manage the data, and facilitate identification of key patterns and concepts.(29) Following initial coding, the team performed directed thematic content analysis through review of the data to summarize recurrent concepts and experiences.(20)

## RESULTS

Six major themes emerged through content analysis as contributors to the uptake of genetic testing for GDD/ID: 1) Caregiver search for answers, advocacy, and empowerment, 2) Healthcare accessibility in a real-life context, 3) Financial strain and insurance coverage, 4) Trust and communication, 5) Racial disparities and discrimination, and 6) Social support and community networks. Representative quotes from each theme are shown in Table 2, and the ways in which each theme connects to the HEIF is outlined in Table 3. Multiple domains within the HEIF emerged consistently, most strongly the patient/caregiver and outer contextual domains.

### Caregivers’ Search for Answers, Advocacy, and Empowerment: “We just wanna make sure that we have all the information we need so that we can give her what she needs.”

Almost all caregivers expressed a desire to understand why their child had GDD/ID, and most reported diagnostic odysseys lasting months to years. During their diagnostic odysseys and beyond, caregivers were huge advocates for their children and played major roles in their children’s diagnostic outcomes. Many had sought second opinions on their child’s diagnosis, with more than one reporting an initial misdiagnosis. Caregivers often completed extensive self-led research, challenged medical opinions when they felt dismissed, and specifically requested certain tests or treatments when necessary, demonstrating a proactive approach to advocating for their child. For most caregivers, empowerment through knowledge was evident, with caregivers feeling more confident in advocating for their child’s care when they had a deeper understanding of their child’s condition and treatment options.

Regarding genetics specifically, most caregivers endorsed positive views on genetic testing as a tool for trying to understand their child’s condition and how best to manage it. Notably, most caregivers did not know what type of genetic testing their child had received, and among those with variants of uncertain significance, there was confusion and uncertainty as to the meaning of the findings. Most, but not all, of those who had received specific genetic diagnoses reported an impact on their child or their family’s medical care, including access to a disease-specific medication, screening for associated congenital anomalies, and testing siblings. Several reported their child’s genetic diagnosis had influenced their family planning decisions. Among those who’d had genetic testing that did not result in a specific diagnosis, no regrets about testing were expressed, and one parent reported relief in knowing their child did not have a fatal genetic condition.

### Healthcare Accessibility in a Real-Life Context: “I just almost wish it was a way where, you know, ‘Okay, well, if you can’t come like we can figure it out,’ or you know?”

Participants encountered various challenges in accessing healthcare services, many of which related to social/“real life” determinants such as transportation, housing stability, rurality, job flexibility, and sibling care needs. These challenges were felt more heavily by those with fewer socioeconomic resources, and they were compounded by organizational/systems-level barriers including lack of specialty providers, long waitlists for evaluations, limited availability of services, and difficulty navigating the healthcare system. Multiple caregivers reported having to quit working to be able to provide the level of care needed for their child and to take them to the necessary appointments/therapies. Several relocated their families to better access healthcare for their child. Many mentioned the need for additional resources to support healthcare navigation and accessibility, and several felt that doctors should have provided this information as part of their medical care.

### Financial Strain and Insurance Coverage: “…it’s not the initial cost, you know…it’s the bill after…”

The extent of financial strain experienced by caregivers of children with GDD/ID varied significantly among participants based on their insurance coverage, employment type and status, and the cost of necessary testing and desired services for their child. Several caregivers reflected on their relative privilege based on having adequate income and/or “good” insurance coverage. Access to insurance coverage was deemed essential, with one parent acknowledging that without insurance, necessary care would have been impossible. Among insurance types, Medicaid was highlighted as providing relatively good coverage, and played a crucial role for many families, covering therapies and medical expenses that would otherwise have been unaffordable. However, navigating Medicaid requirements and limitations also posed challenges, with some parents having to advocate vigorously for coverage. For those with private insurance, multiple reported the burden of insurance deductibles and ongoing financial strain due to medical bills. Having secondary Medicaid insurance coverage and/or a developmental disability waiver for services were reported to ease some of this strain.

For many participants from two-partner families, due to the level of care needed by their child, only one partner was able to work, which contributed to financial strain. Job loss also posed significant challenges, as seen when one participant’s partner was laid off. Conversely, those with flexible, supportive, well-paying jobs noted that healthcare was relatively accessible for them, though many still expressed frustration and challenges including denial of coverage for recommended treatments, delays in approval, and limitations on options.

### Trust & Communication: “The most challenging? Just to get people to listen.”

Trust in the healthcare system and providers varied among participants, with some expressing satisfaction and gratitude for care received, while others voiced frustration, skepticism, and a sense of being unheard or dismissed. Effective communication and empathy from healthcare providers were valued by participants, while deficiencies in communication negatively impacted healthcare experiences. Participants highlighted the role of transparency in building trust and advocated for clear and honest communication about diagnoses, treatment plans, and potential risks or limitations.

They also emphasized the importance of feeling valued and involved in decision-making processes regarding their child’s care and stressed the need for providers to demonstrate cultural humility during these discussions. Those who felt actively engaged and informed about their treatment options reported higher levels of trust and satisfaction with healthcare providers. Participants also highlighted the significance of continuity of care, noting that consistent communication and follow-up from providers contributed to a sense of trust and confidence. In particular, those with strong relationships with primary care providers reported relatively easier journeys to diagnosis and management for their children.

Concerning genetic testing, several participants reported instances where healthcare providers downplayed caregivers’ interest in genetic testing or disregarded their family history, leading to frustration and a sense of being overlooked. These individuals emphasized the importance of healthcare providers acknowledging and addressing their questions and apprehensions regarding genetic testing, as well as respecting their autonomy in decision-making related to genetic screening and counseling. Several highlighted the consequential impact of conversations in which a genetic diagnosis was disclosed, emphasizing the need for empathy, clarity, and humility.

### Racial Disparities & Discrimination: “…it is what it is because I’m Black. And it’s not friendly at all.”

Concerns about racial discrimination within the healthcare system emerged as a central theme in participants’ narratives, revealing pervasive disparities in treatment and access to care based on race. Participants who self-identified as Black/African American described instances where they felt marginalized, dismissed, or mistreated by healthcare providers due to their race. Additionally, one White parent shared an experience of observing their child’s Black birth mother receive substandard care, highlighting the stark contrast in quality of care based on race.

Similarly, Black/African American participants discussed the impact of racial stereotypes and biases on their interactions with healthcare professionals. Some recounted feeling judged or overlooked because of their race, leading to dismissive attitudes by healthcare providers and inadequate care. Several expressed frustration with the lack of cultural humility among healthcare providers, citing instances where their cultural values and beliefs were disregarded or misunderstood. For example, one participant described feeling pressured to conform to Western medical practices, despite advocating for holistic approaches rooted in their Haitian heritage. This disconnect between patients’ cultural backgrounds and the healthcare system’s norms further perpetuated feelings of marginalization and mistrust among Black/African American participants.

### Social Support and Community Networks: “And unfortunately, whenever your child has a disability, there isn’t a great community.”

Social support networks played a crucial role in the lives of families raising children with disabilities, impacting family dynamics, well-being, and access to services. Among the experiences shared, it’s clear that the strength of these social networks varied significantly. Some families were fortunate to have a robust community willing to lend a helping hand during times of need, providing practical assistance and emotional support. A few identified their genetic diagnosis as the impetus for community connection. However, others reported feeling isolated due to their child’s disability, unable to find recognition and understanding among community members.

Social determinants including community affluence/poverty, race/ethnicity, and rurality influenced participants’ perceptions of community/social support. Affluent areas with higher levels of school-based support and specialized programs for children with disabilities provided a sense of community to participants living there, while high-poverty areas were perceived as less supportive. Racial discrimination further compounded challenges faced by several families seeking support. Paraphrasing one participant, societal biases and prejudices can intensify the already daunting task of caring for a child with a disability, particularly for parents perceived as Black/African American.

### Caregiver-informed future outreach sites

**Figure 3:**
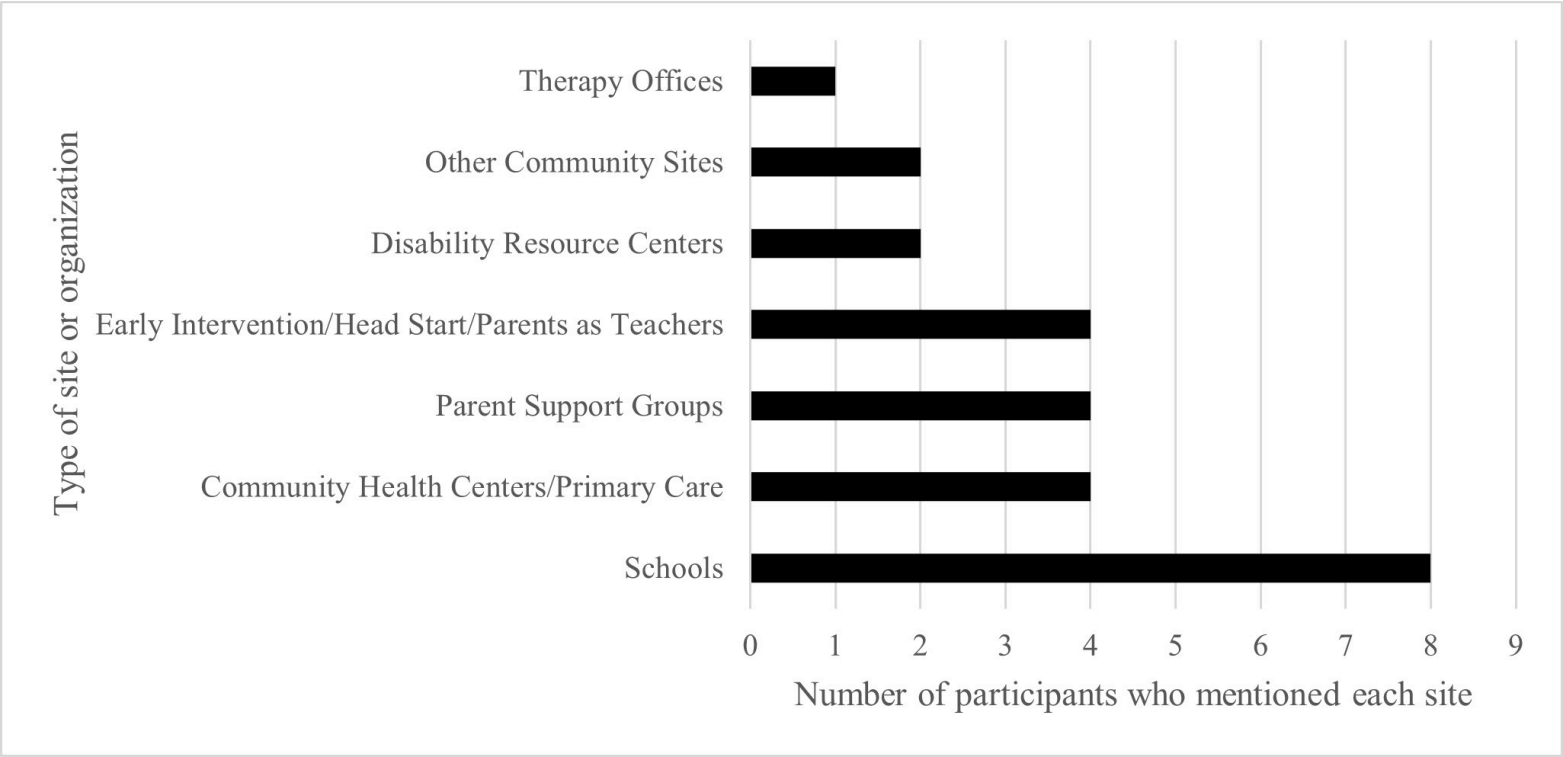
Caregiver-Recommended Sites to Reach Families of Children with GDD/ID: At the end of the semi-structured qualitative interviews, participants were asked about ways in which researchers could try to reach families of children with neurodevelopmental disorders who are not connected with the medical system. This bar graph demonstrates the number of times each type of site/organization was mentioned by a unique participant.

To wrap up the interviews, participants were asked about ways in which health providers/researchers could best reach out to families of kids with developmental disorders who may not engage with the healthcare system (Fig. 3).

## DISCUSSION

Thematic content analysis of semi-structured qualitative interviews conducted among 18 diverse caregivers of children with GDD/ID led to the identification of six themes reflecting experiences, perspectives, and values related to the diagnostic odyssey, genetic testing, and healthcare in general.

The theme of caregivers’ search for answers, advocacy, and empowerment echoed previously reported studies of experiences with the diagnostic odyssey and genetic testing for children with undiagnosed disorders, reflecting caregivers’ extensive efforts to understand and advocate for their child’s needs, and the inherent value of etiologic information. (12, 30) As healthcare providers, it is important to validate caregivers’ desire to understand reasons for their child’s GDD/ID, and to empower caregivers through shared decision-making and medical education tailored specifically to their child. With appropriate counseling and consent, early use of broad genetic testing should be employed to aid these goals.

The vast majority of caregivers experienced challenges navigating the healthcare system, reflected by the theme of healthcare accessibility in a “real life” context. The degree of challenges experienced and the ability to overcome them was greatly influenced by socioecological factors. This was in line with prior studies showing social determinants of health-related disparities in age at diagnosis,(22) access to medical specialists,(31) uptake of genetic testing,(13) and therapy service usage(32) among children with neurodevelopmental disorders in the United States, but provided rich detail into specific barriers faced. Aside from systems-level improvements in workforce numbers and the efficiency of diagnostic processes, targetable strategies to improve healthcare access, particularly for low-resourced individuals, include offering transportation, local outreach clinic sites, flexibility of appointment scheduling/rescheduling, childcare for siblings, and use of culturally competent community-based healthcare navigators.

Financial strain and insurance coverage as a theme qualified the significant known financial impacts of caring for a child with a neurodevelopmental disability, which relates to both decreased parental work hours and costs of medical care not covered by insurance.(33, 34) Dedicated efforts by providers to improve families’ awareness of available financial and insurance supports are needed to assist families in optimizing use of available resources.

Trust and communication were tightly intertwined and bidirectional for caregivers. Particularly poignant was the communication surrounding life-changing diagnoses, abnormal test results, and treatment decision-making. To align with best practices, we recommend disclosure of positive results at a scheduled in-person or telehealth visit, with personalization of the information discussed, provision of accessible resources for self-led education, linkage to family organizations, and a clear outline of next steps.(35)

Racial disparities and discrimination was reflected on by several Black/African American participants, who recounted experiences of biased and culturally insensitive care, providing valuable narratives reflective of the pervasive racial inequities in the United States healthcare system.(36) Cultural humility training for healthcare providers, increased representation of individuals who identify as Black/African American in the healthcare workforce, and systemic reforms to address disparities in income, education, and community-based services are essential steps that should be taken to foster a more inclusive and supportive healthcare environment for Black/African American patients and families.

Social support and community networks were perceived as valuable assets but varied markedly, often in association with social determinants such as rurality, race/ethnicity, and neighborhood affluence. There is a need for inclusive and empathetic community spaces that transcend racial and geographical divides and provide unwavering support to all families, regardless of background. Healthcare provider identification of families with low levels of social/community support should automatically prompt a referral to social work to connect the family with resources.

Each of the six core themes reflect key constructs across domains of the Health Equity Implementation Framework. In particular, factors influencing access to healthcare and genetic testing for children with GDD/ID were most strongly tied to the outer (societal/structural) contextual domain. Caregivers’ narratives yielded themes of financial strain and insurance coverage and racial disparities and discrimination, which vividly illustrated the systemic and structural determinants of care in the outer contextual domain. We found that themes spanning other domains of the HEIF were also key drivers of implementation, from the perspective of parents/caregivers. The inner (local and organizational) contextual domain corresponded to themes of healthcare accessibility in a “real life” context and social support and community networks, reflecting how community-level resources and institutional structures shape families’ ability to navigate care. The clinical encounter domain encompassed trust and communication as well as racial disparities and discrimination, describing how interpersonal experiences impact caregivers’ confidence in and engagement with the healthcare system. Finally, the innermost levels of the framework, patients and families and genetic testing, were reflected in the theme search for answers, advocacy, uncertainty, and empowerment. This emphasizes how individual-level factors can intersect with broader contextual influences across the diagnostic odyssey.

Given the saliency of the lengthy process characterized as a diagnostic odyssey, it is important to consider implementation determinants from the lens of implementation speed or pace. The Framework to Assess Speed of Translation of health innovations (FAST) provides a heuristic for understanding the identified HEIF domains and constructs in terms of speeding or slowing the process of implementation.(37) For instance, identified innovation-level characteristics (e.g., perceived utility of genetic testing) may be considered an “accelerator” whereas recipient-level factors (e.g., financial strain) may be considered an “inhibitor”. In addition, policies in the outer context (e.g., variable insurance coverage) and inner context (e.g., clinic challenges with appointment cancellation/late policies) may contribute to critical “leaks” that further drive delays in the odyssey. Finally, long waitlists and more general limits in healthcare accessibility manifest as factors that slow the “rate of flow” in the research-to-practice gap, impacting the extent to which patients and caregivers can realize the benefits of evidence-based innovations such as genetic testing.

There are several limitations to our study. First, our study population represents a convenience sample of patients seen in a tertiary care academic pediatric neurology clinic. Individuals identifying as Black/African American were over-represented compared to the demographics of the hospital’s catchment area. The patients about whom caregivers were interviewed tended to have moderate-to-severe GDD/ID and high levels of comorbidities including epilepsy. Thus, the perspectives and experiences of our study population may not be reflective of the larger GDD/ID population. Participants’ recommendations for sites to reach families who do not engage with the medical system offer several avenues for potential access including schools, community organizations/health clinics, and online support groups, which may inform future research activities. Second, as a qualitative study, there is potential for analyst biases in coding and generation of themes. Reflexivity was used throughout the study process, and triangulation of results with future quantitative data is planned.

In summary, six major themes emerged from interviews with caregivers of children with GDD/ID regarding the diagnostic odyssey and genetic testing. There was marked variation in the lived experiences of caregivers, much related to social determinants including insurance type, job security, ability to relocate, rurality, family structure, and racism, but a significant degree related to barriers and facilitators of provider/organizational. Improving cultural humility, resource availability and awareness, efficiency of diagnostic processes, and insurance coverage policies are initial steps toward improving the quality and equity of the diagnostic odyssey for GDD/ID.

## Supplementary Material

This is a list of supplementary files associated with this preprint. Click to download.

• AdditionalFile1InterviewGuide.2025.12.15.docx

• AdditionalFile3Codebook2025.12.15.docx

• AdditionalFile2COREQchecklist2025.12.15.docx

## Figures and Tables

**Table 1:** Demographic & Clinical Characteristics of Interview Participants & Patients

PARTICIPANT (CAREGIVER) CHARACTERISTICS (N = 18)		
Recruitment Method	Flyer responder	10 (55.6%)
	Recruitment call	8 (44.4%)
Relationship to Patient	Biological mother	16 (88.9%)
	Adoptive mother	1 (5.6%)
	Biological aunt (guardian)	1 (5.6%)
Race/Ethnicity	Black/African American	6 (33.3%)
	White	12 (66.7%)
Marital Status	Living with partner	2 (11.1%)
	Married	10 (55.6%)
	Never married/Single	4 (22.2%)
	Widowed	1 (5.6%)
	Declined	1 (5.6%)
Highest Level of Education	High school degree or less	5 (27.8%)
	Some college	3 (16.7%)
	Bachelor's degree or higher	9 (50.0%)
	Declined	1 (5.6%)
Area Deprivation Index (National Quintile)	1st (most privileged)	0 (0.0%)
	2nd	4 (22.2%)
	3rd	4 (22.2%)
	4th	5 (27.8%)
	5th (most deprived)	5 (27.8%)
Rural/Urban*	Rural (micropolitan or small town)	2 (11.1%)
	Urban (metropolitan)	16 (88.9%)
Government Benefits Received	Supplemental security income, WIC, and/or food stamps	6 (33.3%)
Met 1 or more criteria for underrepresented in research	≥1 of the following: non-White race/ethnicity, high school degree or less, most deprived ADI quintile, rural	10 (55.6%)
PATIENT CHARACTERISTICS (N = 18)		
Age (at interview date)	< 12mo	1 (5.6%)
	1y0mo to 5y11mo	8 (44.4%)
	6y0mo to 11y11mo	5 (27.8%)
	12y0mo to 17y11mo	4 (22.2%)
Race/Ethnicity	Black/African American	7 (38.9%)
	Multiracial (Black/AA & White)	1 (5.6%)
	Multiracial (Hispanic & White)	1 (5.6%)
	White	9 (50.0%)
Insurance Type	Medicaid only	6 (33.3%)
	Private insurance only	4 (22.2%)
	Private insurance + Secondary Medicaid	7 (38.9%)
	Tricare	1 (5.6%)
Clinical Diagnoses	GDD/ID	18 (100.0%)
	ASD	5 (27.8%)
	Epilepsy	12 (66.7%)
Prior Genetic Testing Type(s)	Karyotype only	1 (5.6%)
	Specific gene testing only	2 (11.1%)
	CMA only	4 (22.2%)
	WES or WES/Mitome only	2 (11.1%)
	Epilepsy panel only	2 (11.1%)
	CMA + CP panel	1 (5.6%)
	CMA + WES or WES/Mitome	4 (22.2%)
	Epilepsy panel + Neuromuscular panel + CP panel	1 (5.6%)
	None	1 (5.6%)
Prior Genetic Testing Outcome	Diagnostic	10/17 (58.8%)

* Rural/Urban status determined based on Rural Urban Commuting Area 2010 codes

**Table 2:** Qualitative Content Analysis Themes & Representative Quotes

Theme	Representative Quotes
**Parental Search for Answers, Advocacy, & Empowerment**	“...yeah, her primary doctor was...and she's great, but she, you know she could admit that what was happening was outside of her scope of things, so she was willing to write us referrals for whatever we wanted to do.” -P3
	“But the genetic testing - we just wanna make sure that we have all the information we need so that we can give her what she needs...that’s why we're doing it. If she's going to have other health issues as she ages...it’s our responsibility to get as much information as we can.” -P6
	“I did my own research and actually reached out to one of the number one doctors who works, who work in this specific, uh, disease at [another medical center], I was able to get her an appointment there to actually meet this doctor and get a better understanding of what’s going on with my child.” -P10
	“...we had to, you know, go out and get a second opinion from someone else to to continue to get more screening in a better evaluation.” -P8
	“ Sometimes I'm interested in [genetic testing]. It’s hard like, you know, you don’t really want to, but then you kind of, you kinda automatically say, well, it’s fine with me. I'm okay with that. It is what it is...I don’t know a lot about my family, so...genetics would help a lot with [knowing] about what happened with him.” -P17
	“I think anything that we can learn about what’s going on is a positive and knowing that there's not something that’s genetically causing it is helpful in understanding future diagnosis, or what else could be going on...so crossing that option, or that that um 'what if' off was very helpful.” -P4
**Healthcare Accessibility in a Real-Life Context**	“I can’t work a day job because of appointments...but having quit...I've got all the time to take her...” -P9
	“.probably just the distance [is most challenging for us]... they don’t have all the specialty doctors that he sees down in our area.” -P2
	“... I do wish it was easier or more understanding for people like me who can’t always make it to appointments. I just almost wish it was a way where, you know, 'Okay, well, if you can’t come like we can figure it out,' or you know?” -P17
	“I mean, we're educated. We're insured. We're very privileged in those ways. And so...it...we have access to things that I know other people don’t.” -P14
	“[Housing instability] ended up um, causing me not to show up for [patient], because I was pre-occupied...I was trying to keep a roof over their head, and trying to do this, and trying to find a place to stay, worried about my safety, worried about my husband's health, worried about like my children.” -P7
	“[Transportation]...is difficult. I honestly feel that like they should be offering that like right at the same time they're making the appointment, it should almost be, 'And did you need transportation for that appointment?' ...I do have a car, but...my car is not like reliable. So, if something happens, I have to cancel.” -P17
	“I think the health care system in general is really hard to navigate, and if...it’s even hard to navigate for me, and I've been doing this for 16 years.” -P13
**Financial Strain & Insurance Coverage**	“I think probably the hardest thing is like insurance deductibles. So, it’s...it’s not the initial cost, you know, because there might not be one initially to get the testing...but then it’s the bill after...” -P5
	“ Prior to my daughter being born... my husband and I both worked ...we were a two-income household. Things were easy. We was able to do whatever we wanted to do and do it freely, but now, with my daughter's medical condition, I can’t really work because she has- she needs...around the clock care...it’s one of those things where financially, yes, it has really affected our family... We have to take our time like, okay, are we paying this bill? Are we buying groceries?” " -P10
	“We've always maxed out our deductible and out of pocket, and it’s not sustainable...luckily we have Medicaid secondary for her now...we have, in the past, had to make certain choices or wait to refill a medication until the proper documentation was in because, um, cause it would be a difference of 6 to 900 dollars for one refill or one afternoon in the ER.” -P9
	“We've never really had to pay for a lot of his stuff because he's had the Medicaid. Fortunately so.” -P1
**Trust & Communication in Healthcare**	“...[getting the genetic diagnosis] was like really, really, heartbreaking because why did it take y'all that long to tell me that she has that? And why you didn’t tell me that it’s a type of–it can’t be curable, but y'all tell me y'all can treat it after this long time?” -P18
	“The most challenging? Just to get people to listen. Getting the doctors to listen.” -P10
	“He left us alone for a couple of minutes, and then came back, and he said, 'Well, folks, you know we knew there was something wrong here.'...and that was it.” -P1
	“I feel like at one point a long time ago someone mentioned [genetic testing], but nothing has been done since the mentioning... I'm willing to do it, but it hasn’t really been...like they just said, 'Have you ever thought of',...but then it’s no like appointment setting for it, it’s not like really, you know what I mean? It’s not like really available. It’s just like a dream, like a cloud.” -P17
	“And that’s when I told my pediatrician that she had stopped rolling...She goes, ['I'm] very concerned that...she has this loss of gross motor skills, so you need to see [the neurologist] sooner.' So, she and he actually like talked on the phone.and he got her in the very next week.” -P6
	“It was just a phone call out of the blue from the doctor. I don’t know if he was very practiced at giving diagnoses, but he told me it was devastating. He...he just used language that wasn’t very...it was very concerning. And he listed off a whole list of of symptoms that weren’t very helpful at that time, like microcephaly and abnormally small feet, just really scary things, you know? And he didn’t really connect us to it– to, you know, any found- there are some great foundations for [specific genetic disorder].” -P8
	“...I was just completely blown away by the way he talked to me, and the way he explained things, and how...he didn’t just take me at my word, he seemed to share the concerns...I have never once felt S-T-U-P-I-D, and I never once felt ignored and I never felt like they were doing something just to make me happy. It was very much...'We see this...I think we need to do A, B and C. Are you okay with that?'” -P6
**Healthcare Inequities, Cultural Values, & Racial Discrimination**	“I don’t want this to sound any kind of way, but it is what it is because I'm Black. And it’s not friendly at all.” -P17
	“I feel like it’s a cultural thing...how you talk to the parent....a lot of doctors are White [or] other ethnicities, and being a Black parent with this baby that has rare, um, rare disease, sometimes I feel like the doctors talk to me as if I'm not educated or I don’t know what’s going on with her [or] how to care for her.” -P10
	“I walked [the social worker] through MyChart, to go and look at the notes of the doctor. And how they were wording everything was as if me and my husband were very dismissive and neglectful.” -P7
	“They don’t have to say anything. It’s how they treat you. The actions behind it. The motives behind it. When you can look right across the hall and see a White family be treated sooo much better, with care, with an authentic smile, with 'Hey, here's a tip, here's a couple tips, you know, off the record', I don’t get that. It’s here you go, get out type of treatment.” -P7
**Social Support & Community Networks**	“[Patient] was a big impetus for us moving to the area...we really tried to focus on places [with] accessibility and available support services... community plays a big part in how we can best support him.” -P14
	“[The genetic diagnosis] opened up a door for us to connect with other families and get a lot of support."-P8
	“And unfortunately, whenever your child has a disability, there isn’t a great community. At least I have felt like that there's not a lot of community out there, um, for support for parents.” - P15
	“[Our child's developmental disorder] has definitely affected our family, because our whole family dynamic, like it, just affects what we, what we can do out with each other, and where we can go, and who can come to our home. Um, but we're all committed to making it work. So, our family is, you know, joyful and healthy, but our world is pretty small.” -P6
	“[Our community], it’s a lot of poverty, it’s a lot of uh, low-income. It’s not, you know, a variety of different people...I feel like that plays a big factor into health.” - P12
	“...we didn’t get any direction when it came to social workers that could help us...like we just went to the neurologist, and that was like it. Like I had no idea there was anything further that could help... make life easier. “ -P13

**Table 3:** Mapping Qualitative Interview Themes to the Health Equity Implementation Framework

Theme	Domain	Determinant(s) of Genetic Testing (GT)
**Caregivers' Search for Answers, Advocacy, and Empowerment**	Recipients: Patients & Families	Values/Motivations: Information, answers/understanding, decreasing uncertainty Health literacy: Caregiver-initiated research and requests for additional testing/referrals
	The Innovation: Genetic testing	Perceived utility: Providing answers and/or reassurance that child does not have a fatal disorder. Ability to connect with others with the same diagnosis. Reproductive decisions for self and other children. Prognostication. Change in medical management for some. Accessibility: All received through specialists, many after multiple referrals/visits.
	Outer context: The healthcare system	Organization & Access: Limited specialists with expertise in specific genetic conditions, lack of awareness among frontline providers, difficulties getting therapies/services needed
**Healthcare Accessibility in a Real-Life Context**	Recipients: Patients & Families	Socioeconomic status: Job, housing, & transportation stability; Educational background
	Outer context: The healthcare system	Location: Difficulties with transportation/traveling distance Policy: Challenges with appointment cancellation/late policies, policies on siblings at appointments/hospital visits
**Financial Strain and Insurance Coverage**	Recipients: Patients & Families	Health insurance status/type: Most with Medicaid experienced few issues with cost/coverage, while those with private insurance reported significant financial hardship related to medical costs and/or inability to work full time job due to patients' medical needs
	Outer context: Payer policies	Payer policies: Variable coverage of genetic testing through payers
**Trust & Communication**	The Clinical Encounter:	Method of disclosing results: Several who received serious diagnoses by phone were distressed and dissatisfied Tone & empathy: A lack of empathy or focus on facts nonspecific to one's individual child were not seen as effective communication Clarity & shared decision-making: For those whose providers communicated their thinking processes clearly and outlined possible next steps, allowing for shared input from caregivers, this was highly valued & promoted trust. Others experienced mistrust when providers proceeded with testing they were unaware of or did not listen to their input/preferences
**Racial Disparities & Discrimination**	Outer context: Systemic racism	Biases & discrimination: Multiple caregivers who identified as Black/African American reported experiences of bias and discrimination when accessing healthcare for their child
**Social Support and Community Networks**	Inner context: Personal support	Caregiver support systems: Many caregivers described limited personal support systems, particularly single parents. The burden of decision-making on single parents was discussed.
	Outer context: Community support	Community support: Several described the experience of parenting a child with a developmental disability as isolating. Those with positive supports through school or genetic disease-specific advocacy groups felt empowered.

## Data Availability

The data presented in this manuscript are available from the corresponding author on reasonable request.
